# Equitable Chick Survival in Three Species of the Non-Migratory Shorebird Despite Species-Specific Sexual Dimorphism of the Young

**DOI:** 10.3390/ani9050271

**Published:** 2019-05-23

**Authors:** Daniel Lees, Tom Schmidt, Craig D. H. Sherman, Grainne S. Maguire, Peter Dann, Michael A. Weston

**Affiliations:** 1 Centre for Integrative Ecology, Faculty of Science, Engineering and the Built Environment, School of Life and Environmental Sciences, Burwood Campus, Deakin University, Geelong, VIC 3125, Australia; tomschmidt@live.com.au (T.S.); mike.weston@deakin.edu.au (M.A.W.); 2 Centre for Integrative Ecology, Faculty of Science, Engineering and the Built Environment, School of Life and Environmental Sciences, Waurn Ponds Campus, Deakin University, Geelong, VIC 3126, Australia; craig.sherman@deakin.edu.au; 3 BirdLife Australia, Suite 2-05, The Green Building, 60 Leicester Street, Carlton, VIC 3052, Australia; grainne.maguire@birdlife.org.au; 4 Research Department, Phillip Island Nature Parks, PO Box 97, Cowes, Phillip Island, VIC 3922, Australia; pdann@penguins.org.au

**Keywords:** lapwing, plover, precocial, survival, growth, sex-ratio

## Abstract

**Simple Summary:**

We aimed to determine whether seasonal brood sex-ratio, sex-biased chick survival, and sex specific dimorphism at hatching or during growth occurs among three species of resident Australian shorebird. Our results describe no sex-bias in chick production, survival or growth rates between sexes for any of the three species studied.

**Abstract:**

Sex-biases in populations can have important implications for species’ social biology, population demography and mating systems. It has recently been suggested that in some shorebirds, sex-specific bias in survival of precocial young may occur. This may be driven by variation in the brood sex-ratio and/or the sexual size dimorphism of young birds, which may influence predator escape capacity. Understanding the survival of young birds remains a significant knowledge gap for many taxa, especially when young birds are mobile and cryptic. Our aims were to estimate the sex-ratio variation in three species of Australian resident shorebird, specifically to determine: (1) whether seasonal brood sex-ratio variation at hatching is occurring, (2) the extent of any sex-biased chick survival, (3) if sex specific dimorphism at hatching or during growth occurs; and, (4) whether escape capacity differs between the sexes. We radio-tracked 50 Masked Lapwing *Vanellus miles*, 42 Red-capped Plover *Charadrius ruficapillus* and 27 Hooded Plover *Thinornis cucullatus* chicks from individual broods, examined the likelihood of hatchlings being male or female based on the hatching date within the breeding season, and compared size at hatching, growth and mortality of chicks of different sexes. There was no sex-bias with the hatching date across the breeding season, nor were there differences in survival or growth rates between sexes for any of the three species studied. In one species, male hatchlings had longer tarsi than females, but this did not result in differential escape propensity or improved survival. In conclusion, the hatching date, survival and growth of chicks from three species of resident shorebird was not influenced by their sex.

## 1. Introduction

Offspring sex-ratios of many animal species are parous as parents invest equally in the sexes [1,2]. In other species females may exhibit skewed brood sex-ratios to maximise their future reproductive potential [3,4]. Fisher [5] suggested under certain circumstances it would be adaptive to bias the investment of offspring in favour of one sex. Future reproductive value of each sex of young may be influenced by: parental traits (e.g., male attractiveness or female condition), environmental characteristics, and the age of first breeding or juvenile survival [4]. Correlations between future reproductive value and maternal sex-ratio adjustment are well documented [6,7,8,9,10], including numerous studies demonstrating variation in the sex-ratio of young produced at hatching across the breeding season. Many key studies have focussed on birds: Whiskered Tern *Chlidonias hybrid* [11], Lesser Kestrel *Falco naumanni* [12], European Shag *Phalacrocorax aristotelis* [13] and Kentish Plover *Charadrius alexandrinus* [8] exhibit skews in their brood sex-ratios at hatching towards males early in the breeding season. In contrast, the Crimson Rosella *Platycercus elegans* [14], Marsh Harrier *Circus aeruginosus* [15] and Peregrine Falcon *Falco peregrinus* [16] exhibit skews in their brood sex-ratios at hatching towards females early in the season. The variation with progression of the breeding season can be attributed to either: (1) early season breeders favouring the sex more likely to successfully breed in the subsequent breeding season [1,17], (2) environmental/resource factors favouring the production of one sex at a given point of the breeding season [11,13]; or (3) over-producing the sex early in the season that will benefit the most from an early start [8,18]. 

Population sex-ratios may also become imbalanced through sex-biased survival of the young. Sex-biased survival of young primarily occurs as an artefact of sexual size and/or growth dimorphism, either through: (1) disparity in nutritional demands leading to increased likelihood of starvation for one sex; or, (2) developmental differences that allow one sex to escape predators more effectively than the other [8,19,20]. The latter is unlikely to be a factor in altricial species, because of their immobile nature and the likelihood that a predator will destroy the entire brood [19]. Precocial young, on the other hand, often spread out, are highly mobile and may scatter when attacked, making it less likely that a predator will destroy the entire brood [8]. Sex-based differences in the size at hatching, growth rate and survival have been demonstrated among precocial species and may result in predators targeting the slower developing sex; causing a sex-bias in survival which favours the faster developing sex [8,18,20,21,22]. 

Shorebird species the world over are in decline [23,24,25]; some of the causes of this decline have already been identified, including; alterations in agricultural practices, the reclamation of feeding habitats, human disturbance and climate change [26,27,28,29,30,31]. In some species of birds, natural variations in sex-ratio dynamics have exacerbated population declines and/or hampered population recovery [32,33]. An understanding of the underlying causes of a sex-ratio imbalance is therefore critical for the conservation of threatened shorebird species [32,34]. Given the decline of shorebirds worldwide [23,24,25], and the troubling examples of how sex-ratio biases stifle population growth in the Kentish, Snowy and Piping Plover *Charadrius melodus* [18,21,22], more studies are required across more species to examine whether seasonal sex-ratio variation at hatching or sex-biased survival of young chicks occurs. Here, we examine whether: (1) seasonal hatchling sex-ratio variation, (2) sex-biased chick survival; and, (3) sexual dimorphism in size at hatching or growth rates occurs among three species of resident shorebirds (Masked Lapwing *Vanellus miles*, Red-capped Plover *Charadrius ruficapillus* and the Vulnerable Hooded Plover *Thinornis cucullatus*). All three species are socially monogamous [35] with the adult sex-ratios of the Masked Lapwing and Red-capped Plover populations being unknown; while the Hooded Plover population is apparently parous [36]. Differences in survival may manifest themselves in a sexually-specific capacity for predator escape [37]. We also examine whether there are differences in the escape capacity between the sexes in Red-capped Plover chicks. We do not expect to find any sex-biased variation among the sexually monomorphic Masked Lapwing or Hooded Plover as, generally, monomorphic and monochromatic species (such as these species) should rarely display variations in sex-ratio dynamics [35,38]. However, we expect to find a degree of seasonal brood sex-ratio variation and/or sex-biased chick survival in the sexually dichromatic Red-capped Plover (whose congeners have exhibited such biases). Specifically, we predict Red-capped Plovers will: produce an excess of male chicks early in the breeding season and an excess of female chicks late in the breeding season; and/or for male chicks to hatch larger and grow faster than female chicks leading to greater male chick survival, predictions that mirror the results documented from the closely related Kentish Plover [8]. 

## 2. Materials and Methods

To find the nests of all three species, systematic searches of known nesting locations occurred throughout the breeding season. Masked Lapwing and Red-capped Plover fieldwork was conducted from June to November 2014, and June 2015 to February 2016, respectively. For the Hooded Plover, this involved volunteer citizen scientists regularly monitoring breeding sites from August 2016 to March 2017 (otherwise Daniel Lees collected all data). Thus, we studied each species in a single, typical, breeding season. The location of study sites and species are provided in Table 1. Where laying date was unknown, eggs were floated to estimate hatch date (and hence laying date) following Liebezeit et al. [39]. Clutch sizes (mean ± standard error [SE]; median) from our study (complete clutches only) were 3.70 ± 0.07 (4) eggs for the Masked Lapwing (411 eggs; *n* = 111), 1.95 ± 0.03 (2) for the Red-capped Plover (142; *n* = 73) and 2.73 ± 0.09 (3) for the Hooded Plover (71; *n* = 26). Clutch sizes did not fluctuate as the breeding season progressed for any species (Appendix A Table A1). Frequent follow up around predicted hatching date allowed most broods to be captured within a day of hatching. However, seven Masked Lapwing broods, seven Red-capped Plover broods and six Hooded Plover broods were found opportunistically and without having previously found the nest. Chicks from these broods had their age estimated through the application of regressions of morphometric characters versus age derived from known-age chicks from previous breeding seasons (i.e., a comparable sample not included in, and thus not confounded with, this study):

$$\mathrm{Masked}\;\mathrm{Lapwing};\;\mathrm{age}\;\left(\mathrm{days}\right)=\frac{\left(71.053+\mathrm{mass}\left[g\right]\right)}{6.225}\;(R^{2}=0.747)\;\mathrm{Red-capped}\;\mathrm{Plover};\;\mathrm{age}\;\left(\mathrm{days}\right)=\frac{\left(\mathrm{tarsus}\;\mathrm{length}\;\left[\mathrm{mm}\right]-19.467\right)}{0.249}\;(R^{2}\;=\;0.884)\;\mathrm{Hooded}\;\mathrm{Plover};\;\mathrm{age}\;\left(\mathrm{days}\right)=\frac{\mathrm{Ln}\;\left(\mathrm{mass}\;\left[g\right]\right)-2.144}{0.094}\;(R^{2}\;=\;0.939)$$

Average brood sizes (mean ± SE; median) were: 3.26 ± 0.12 (3) chicks for Masked Lapwing, 1.86 ± 0.05 (2) chicks for Red-capped Plover and 1.81 ± 0.14 (2) chicks for Hooded Plover. Chicks from all three species hatch more-or-less synchronously and were only captured after all eggs in a nest had hatched (usually in the nest or within ~50 m), chicks were captured by hand and morphometrics were measured (mass, tarsus length, tarsus plus toe length, bill length and head plus bill length). These measurements reflect size and condition, and in the case of tarsus length, possibly escape capacity (precocial shorebirds run to escape). A small ~50 µL blood sample was taken from the tarsal vein. Masked Lapwing and Red-capped Plover blood samples were sexed following Fridolfsson and Ellegren [40]; Hooded Plover samples were sexed commercially by DNA solutions^TM^. All chicks were successfully sexed and assignments were confirmed without exception by observations of mature Red-capped Plover (sexually dichromatic), and for all species known sex partners and copulatory position. One chick within a given brood was randomly selected for radio-transmitter attachment; the selected chick had a radio-transmitter (Table 1; Appendix A Table A2) attached using the ‘glue-on’ method of backpack radio-transmitter attachment (after Göth and Jones [41]). Survival of chicks with and without trackers was equivalent ([42], Lees et al. in review [43]), as was the number of male and female chicks fitted with transmitters (Masked Lapwing, 28 males and 22 females, exact binomial test, 95% CI = 0.413–0.700, *p* = 0.480; Red-capped Plover, 23 males, 19 females, 0.387–0.702, *p* = 0.644; Hooded Plover, 15 males, 12 females, 0.353–0.745, *p* = 0.701). Chicks fitted with a transmitter were then ringed with a unique ABBBS metal band on the left tarsus to aid identification, while its siblings were ringed on the right tarsus. Prior to releasing Red-capped Plover chicks; and only if conditions were appropriate (mild temperatures, no predators), the propensity of each chick to escape was quantified. The “escape trial” (adapted from Martín et al. [44]) was conducted by placing the chick on a flat section of ground. A circle two metres in radius and away from any cover was demarcated, and a stopwatch was used to time how long (up until two minutes) it took for the chick to flee past the perimeter. These chicks were newly hatched at approximately one day old; young shorebird chicks combine hiding (immobility) and active (running) responses. Here, we use a holistic measure of chick escape, and acknowledge we do not directly measure locomotory capacity. 

We radio-tracked chicks diurnally, determining if the radio-tagged chick and its siblings were alive or had died [45]. The chick and brood location was determined by visually identifying the radio-tagged chick from afar using optical equipment. When radio-transmitters were identified as having fallen off, the radio-tagged chick was re-captured and a new radio-transmitter was attached. While radio-tracking Masked Lapwing chicks, they were assumed to have died and monitoring of the brood ceased, when signals were lost and parents (identified by previous marking efforts) were located on three consecutive occasions without the radio-tagged chick. If the carcass of the radio-tagged chick was found, radio-tracking ceased immediately. Locating untagged and relatively large and mobile Masked Lapwing broods in the urban matrix, consisting of multiple private properties used by each brood, proved unmanageable. In contrast, for Red-capped Plovers and Hooded Plovers, if the tagged chick died, follow up of the sibling/s was achieved via the observation of parents flagged with unique alpha-alpha leg flags, and monitoring ceased when all chicks within a brood had died or fledged. Alternatively, the chick and/or its sibling/s were deemed to have fledged when it was observed to be flying strongly or had reached fledging age (35 days old for Red-capped Plover and Hooded Plover; 45 days old for Masked Lapwing) [35,42,46]. For all three species, parental identity (even in the absence of chicks) was confirmed through the observation of uniquely engraved alpha-alpha leg flags placed on adult birds throughout these and previous breeding seasons. 

Where possible, just prior to fledging, Masked Lapwing and Red-capped Plover chicks were re-captured for the assessment of a second round of morphometrics. To minimise disturbance to Hooded Plover chicks (a disturbance-sensitive species [47]), we elected not to recapture chicks as they approached fledging. As the Hooded Plover chicks that were being radio-tracked were not recaptured prior to fledging we used morphometric data from 37 known age, known sex chicks at least 10 days old captured between 2011 and 2018 as part of BirdLife Australia’s ‘Beach-nesting Bird’ recovery program. 

### Statistical Analysis

Data for each species was analysed separately, with each brood treated as an independent data point. Exact binomial tests (for each species) examined whether the overall hatching sex-ratios varied from parity. Three separate binomial logistic Generalized Linear Mixed Models (GLMMs) investigated whether chicks were more likely to be male (or female) as the breeding season progressed; the binomial status of sex was a response variable (0 = female, 1 = male), day of the year (number of days since the first nest from each species was predicted to have been laid; representing the progression of the breeding season) was used as a fixed continuous factor while brood identity was used as a random factor. 

Masked Lapwing brood monitoring ceased after the death of the radio-tagged chick, and lapwings usually had broods of four chicks; as such when siblings died, we did not know their exact identity. Hence, only the 50 radio-tagged chicks (not their siblings) were included in analysis. For the other two species, it was possible to diligently follow up the identity of deceased siblings of the radio-tracked chick. This was aided by smaller brood sizes for Red-capped and Hooded Plovers, two and three respectively [35]. Red-capped and Hooded Plover brood data was complete; thus, survival data from radio-tagged chicks and their un-tracked siblings was included. To determine if the sex of each chick influenced survival, data from broods containing radio-tagged chicks were analysed using three separate Cox proportional hazard regressions (one per species). Survival analyses were run with the binomial status of all chicks at the time of death or fledging as a response variable (0 = alive, 1 = deceased), the sex of the chick and brood size at hatching as fixed factors, while brood identity was used as a random effect. Brood identity was not included in the survival analysis of Masked Lapwings as only radio-tagged chicks from different, independent, broods were included in this analysis. We used three Kaplan-Meier curves to visualise the sex-based survival probability of chicks among all three species. Two Masked Lapwing chicks were removed from analysis of hatching morphometrics, as they were not able to be captured on the first attempt and were instead opportunistically encountered in the subsequent days. We acknowledge that broods captured opportunistically may be incomplete and have suffered mortality prior to capture, however this was infrequent (16.8% of all broods were opportunistically captured) and not considered to unduly influence our findings. 

To identify potential relationships between morphometric measures at hatching, morphometric characters were reduced using principal component analysis (PCA) with varimax rotation [48]. Variables included in the PCA for each species were: mass, tarsus length, tarsus plus toe length, bill length and head plus bill length. Principal components for each species and their explanatory value are described in Table 2. For each species, one (Hooded Plover) or two (Masked Lapwing and Red-capped Plover) principal components were extracted. We interpret these components by examining positive component loadings of ≥ 0.6 for the rotated solution. The principal components can thus be interpreted as: Masked Lapwing ‘mass and tarsus length’ (mass, tarsus and tarsus plus toe length), Masked Lapwing ‘head size’ (bill and head plus bill length), Red-capped Plover ‘head size’ (bill and head plus bill length), Red-capped Plover ‘mass and tarsus length’ (mass, tarsus and tarsus plus toe length) and Hooded Plover ‘structural size’ (all measurements excluding mass) (Table 2). While the PCAs produce uncorrelated metrics of body form, we also present each (correlated) morphological character separately, as this can identify specific single-character differences. Bivariate correlations with a magnitude ≥ 0.5 were: tarsus and tarsus plus toe, and head plus bill and tarsus plus toe (all species); and for Masked Lapwing and Red-capped Plover only, tarsus and head plus bill, and bill and head plus bill. 

Separate Linear Mixed-effect Models (LMMs) of each morphological character and principal component determined if chicks were sexually size dimorphic at hatching. These separate LMMs used mass, tarsus length, bill length, head plus bill length, body condition at hatching and the extracted principal components as response variables. Chick sex was a predictor variable and brood identity was a random effect. Broods captured opportunistically were omitted from this analysis. Separate LMM’s determined if male chicks grew faster or slower than female chicks. These LMMs used change in mass and tarsus length between captures as response variables (again, separate models), chick sex and chick age were used as predictor variables, while brood identity was a random effect. Chicks initially captured and whose age was not known had their age assigned using the aforementioned linear models of known age chicks not part of the study. We used a Scaled Mass Index (SMI; a mass length relationship [49]) to characterise body condition:

$$SMI=M_{i}{\left[\frac{L_{0}}{L_{i}}\right]}^{{}^{b}SMA}$$

where M_i_ and L_i_ are body mass and linear body measurements (chick weight and tarsus length in this study) of individual *i*. While *L*_0_ is an arbitrary value of length and *^b^SMA* is a scaling exponent estimated by the SMA regression of mass on length. Male Red-capped Plover hatchlings had significantly longer tarsi than females; as such it was inappropriate to use tarsus length for the calculation of scaled mass index in this species and we instead used a different structural measurement i.e., head plus bill length (which did not vary between the sexes). 

Finally, a binomial logistic Generalized Linear Mixed Model (GLMM) tested whether the propensity to escape differed between male and female Red-capped Plover chicks. The anticipated continuous measure did not eventuate, instead individuals fled (*n* = 9) or hid (*n* = 29). Thus, our measure indicated propensity to engage in active escape, but does not measure locomotory capacity (e.g., speed) between the sexes. This GLMM was run with propensity to escape as a response variable (0 = hid; 1 = fled), sex as a predictor variable, while brood identity was a random effect. Chick age was not included in the GLMM, as all chicks that undertook the escape trial were newly hatched. 

Statistical tests were conducted in R [50] with the Cox proportional hazard regression and Kaplan-Meier curve generated in the package ‘survival’, the ordinal regression (Appendix A Table A1) in the package ‘ordinal’ and the LMM’s/GLMM’s conducted in the packages ‘nlme’ and ‘lme4’ [51,52,53,54]. PCAs were conducted in IBM SPSS for Windows [55]. 

## 3. Results

We found no evidence for overall sex-bias at hatching in any of the study species (Table 3). Young of all three species were no more or less likely to be male (or female) as the breeding season progressed (Table 4). 

**Figure 1:**
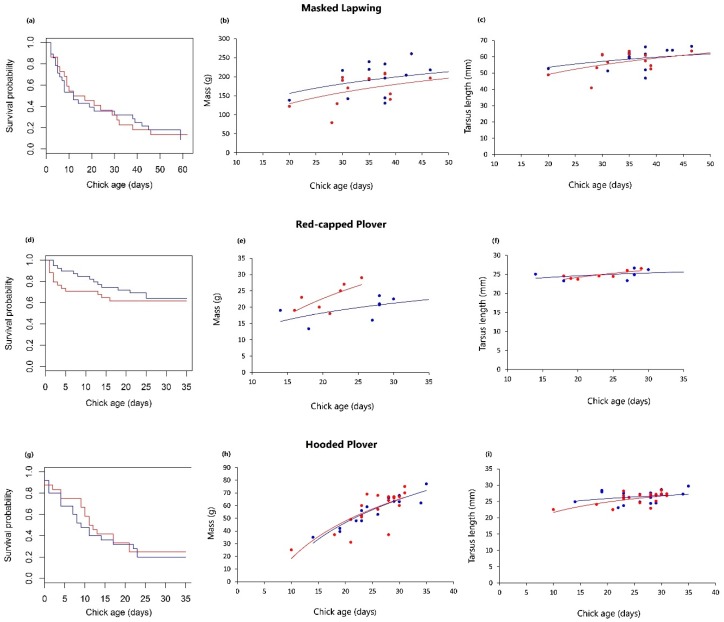
(**a**) Kaplan-Meier survival curve comparing the survival of male and female Masked Lapwing chicks. (**b**) Increase in mass between the sexes in Masked Lapwing chicks (raw data). (**c**) Increase in tarsus length between the sexes in Masked Lapwing chicks (raw data). (**d**) Survival of male and female Red-capped Plover chicks. (**e**) Increase in mass between the sexes in Red-capped Plover chicks (raw data). (**f**) Increase in tarsus length between the sexes in Red-capped Plover chicks (raw data). (**g**) Survival of male and female Hooded Plover chicks. (**h**) Increase in mass between the sexes in Hooded Plover chicks (raw data). (**i**) Increase in tarsus length between the sexes in Hooded Plover chicks (raw data). Blue curves and points = male chicks, red curves = female chicks.

There was no difference in mass, tarsus plus toe length, bill length, head plus bill length, body condition or any principal component at hatching between the sexes for any of the species, nor were there differences between the sexes in tarsus length at hatching for Masked Lapwing or Hooded Plover (Table 5; Appendix A Table A3). However, male Red-capped Plover chicks hatched with significantly but slightly longer tarsi than females (mean ± SE; male, 19.47 ± 0.13 mm; female, 19.08 ± 0.14 mm; Table 5), although both sexes realise the same growth rate in tarsi (Table 6; Figure 1). The difference in tarsi length did not translate into a different propensity to escape (C ± SE = 0.003 ± 0.826; *Z* = 0.004; *p* = 0.997). Otherwise, there was no difference in tarsus length growth or growth in mass between sexes for any of the study species (Table 6; Figure 1). 

Although survival between species differed substantially; male and female chicks from all three species had equivalent survival (Table 7; Figure 1). Brood identity was significantly associated with the survival of Red-capped and Hooded Plover chicks (Table 7). 

## 4. Discussion

Overall biases in brood sex-ratio have been reported in many species, especially those that display sexual size dimorphism (that may invest more in members of the sex that is the least costly to raise [56]). Brood sex-ratio bias also has occasionally been described in species that do not display sexual size dimorphism [57]. These are generally theorised to be maternal manipulations of brood sex-ratio in response to food supply or maternal condition (e.g., references [13,32,58]). This study detected parous sex-ratios at hatching for three Australian resident shorebirds, one of which is sexually dimorphic and dichromatic. This parity of sex-ratio at hatching aligns with numerous other studies on plovers [8,59,60,61]. Even species displaying sexual size dimorphism such as the Red-necked Phalarope *Phalaropus lobatus* may still produce clutches at parity while variation occurs in another aspect of breeding, such as sex-biases in egg size [62]. Red-capped Plovers are dichromatic and have a significant yet slight bias in tarsus length size favouring males (this study; Lees et al. unpublished data [35]). Masked Lapwings and Hooded Plovers are monomorphic and monochromatic; as such there is likely no evolutionary basis for females to invest in one sex over the other [35].

We detected no seasonal sex-ratio variation at hatching among broods of the sexually monomorphic and monochromatic Masked Lapwing and Hooded Plover, or the sexually dimorphic and dichromatic Red-capped Plover. In particular we expected some degree of seasonal brood sex-ratio variation in the Red-capped Plover, as has been detected for northern hemisphere congeners [8,60]. The Kentish Plover transitions (at least in some populations) from the production of male-biased broods at hatching early in the breeding season to female-biased broods late in the breeding season [8]. This transition is thought to optimise lifetime reproductive success by conferring an advantage to male chicks. With an adult sex-ratio of 0.860 and hence extreme competition for mates, the added time to mature enabled by earlier hatching may better facilitate males breeding in the next season [8,18]. Similarly, the closely related Snowy Plover also exhibits seasonal brood sex-ratio variation at hatching, but in contrast the Snowy Plover produces an excess of males at the start and end of the breeding season and an excess of females in the middle of the breeding season [59]. This bimodal variation can be attributed to the greater stability of food resources at the start and end of the breeding season as extreme temperatures evaporate surface water and cause food resources to become scarce at the height of the breeding season. Because male Snowy Plovers defend breeding territories during the breeding season and males hatch with longer tarsi, the authors theorise that the future fitness benefits of a more stable food resource, secured by territory establishment, are potentially greater for male chicks than for females [60]. Our study aligns with a more recent study of a population of Kentish Plover in China that did not display any seasonal brood sex-ratio variation [63]. Thus, across and even within species, seasonal brood sex-ratio variation is plastic and has the capacity to fluctuate if young of one sex are advantaged by prevailing environmental conditions and/or the adult sex-ratio. Indeed, it would be interesting to determine whether Red-capped Plover populations occupying ephemeral habitats (in Australia’s interior) display seasonal brood sex-ratio variation. 

The equitable survival to fledging between the sexes which we describe may help explain the parous adult sex-ratio of the Red-capped Plover study population (0.546; Lees et al. unpublished data; the only species studied for which adult sex ratio is readily attainable). After hatching, precocial young spread out within flexible home ranges, within which they scatter in response to predators or parental alarm, making it less likely that a predation event will destroy the entire brood [8,19]. It is therefore surprising that our survival analysis revealed brood identity to be significantly associated with survival of Red-capped and Hooded Plover chicks. Whether this result is an artefact of parental quality (i.e., capacity to defend chicks), the presence of chick predators in some home ranges but not others or the quality of the territory itself is unknown. Soon after hatching, precocial and nidifugous chicks suffer substantial mortality [64,65], a result mirrored for all species studied here (Figure 1). Among all three species we studied, the greatest mortality occurred within the first week of hatching. This has been previously documented for Hooded Plover [66] and briefly in the Red-capped Plover [45] but not the Masked Lapwing. Spatial variation in sex-specific survival and causes of mortality warrant further investigation, as even with radio-tracking, the causes of chick mortality remain largely unknown (Lees et al. unpublished data, [42]). 

For the Masked Lapwing or Hooded Plover, neither sex hatched with an advantage in size (including our extracted principal components) or body condition; both species are sexually monomorphic as adults (Lees et al. unpublished data; [35]). However, male Red-capped Plovers had longer tarsi (but did not otherwise differ in size at hatching; an almost identical pattern reported for at least some Kentish and Snowy Plover populations [8,20,60]). Longer tarsi could mean an enhanced capacity to escape, as pre-flight chicks rely on running and hiding to avoid predators [8]. However, this study shows that longer tarsi do not always influence the propensity to escape or chick survival. Theoretically longer tarsi could provide a foraging advantage allowing males to cover more distance with less energetic expenditure; but this seems unlikely as we did not detect a difference in growth or survival between the sexes. Interestingly, this difference in tarsus length at hatching (males with an average tarsus length 0.39 mm longer than females) translates into a sex difference in adult tarsi (adult males have an average tarsus length 0.44 mm longer than females; Lees et al. unpublished data). Thus, with no difference in tarsus length growth between the sexes, males have slightly longer tarsi throughout life. We note that this difference in tarsus length is only 2% of total tarsus length at hatching, and may not be sufficient to advantage either sex. 

Many species display a sex-bias in egg laying order (one sex for example being more likely to be produced in first laid egg/s) along with a decrease in volume in each subsequent egg [67,68]. This increased likelihood that one sex will be produced earlier in clutches and in larger eggs may conceivably manifest itself in a size difference between sexes at hatching [67,68]. Whether a sex-bias in egg laying order occurs in our study species remains unknown, as chicks often hatch within hours of each other and determining which chick came from which egg was not possible (Lees et al. pers. Obs.). 

## 5. Conclusions

We found no evidence of seasonal brood sex-ratio variation at hatching or sex-biased survival of young in three species of the non-migratory shorebird. This is despite slight sexual dimorphism in the young of one of these species, a result which contrasts with those reported for congeners [8,18,21,22]. Discrepancies between the sex-ratios at hatching, fledging and the adult sex-ratio may occur via sex-biases in immature and/or adult survival [22]. Future research on sex-bias of flying age birds are therefore required to fully assess the role, if any, that sex-bias may play in shorebird populations.

## Figures and Tables

**Table 1:** Study species, number of chicks/broods studied and the habitats and sites where radio-tracking occurred.

Species	Radio-Tracked Chicks (N)	Un-Tracked Siblings (N)	Total Chicks (N)	Habitats	Study Area (Centroid)
Masked Lapwing	50	106	156	Suburban and rural parts of fox-free Phillip Island	38°29′06″ S, 145°13′47″ E
Red-capped Plover	42	36	78	Disused salt works, western Port Phillip Bay, Melbourne	37°53′56″ S, 144°47′33″ E
Hooded Plover	27	22	49	Ocean beaches of central, southern Victoria	38°29′14″ S, 144°58′13″ E

**Table 2:** Component scores derived from a principal component analysis with varimax rotation of five morphometric variables measured to characterise hatchling size. Component scores ≥ 0.60 were used to interpret the principal components (PC) and are emboldened. Percentage of variation explained by each component is presented in brackets.

Variables	Masked Lapwing PC1 (‘Mass and Tarsus Length’; 42.8%)	Masked Lapwing PC2 (‘Head Size’; 37.6%)	Red-capped Plover PC1 (‘Head Size’; 40.8%)	Red-capped Plover PC2 (‘Mass And Tarsus Length’; 39.3%)	Hooded Plover PC1 (‘Structural Size’; 49.6%)
Mass	**0.814**	−0.012	−0.232	**0.872**	0.558
Tarsus length	**0.807**	0.382	0.464	**0.775**	**0.718**
Tarsus plus toe length	**0.815**	0.341	0.498	**0.711**	**0.792**
Bill length	0.064	**0.949**	**0.903**	−0.073	**0.646**
Head plus bill length	0.402	**0.846**	**0.841**	0.304	**0.780**

**Table 3:** Exact binomial tests of the sex-ratio at hatching for Masked Lapwing, Red-capped Plover and Hooded Plover (separate models). CI is confidence interval.

Species	Lower 95% CI	Upper 95% CI	*N*	*p*-Value
Masked Lapwing	0.396	0.574	130	0.793
Red-capped Plover	0.386	0.645	62	0.899
Hooded Plover	0.374	0.663	50	0.888

**Table 4:** Generalized linear mixed model (GLMM) results examining the influence of progression of the breeding season on the brood sex-ratio at hatching (separate models for each species). C ± SE = coefficient ± standard error.

Species	C ± SE	*z*-Value	*p*-Value
Masked Lapwing	−0.001 ± 0.010	−0.116	0.907
Red-capped Plover	−0.002 ± 0.007	−0.338	0.735
Hooded Plover	−0.314 ± 0.382	−0.823	0.410

**Table 5:** Linear Mixed-effect Models comparing each character (chick mass, tarsus length, bill length, head plus bill length, body condition) and principal components at hatching between the sexes of each species. In all models, data from male chicks was the reference variable. Significant values are emboldened. PC = Principal Component, Df = degrees of freedom.

Species.	Metric	C ± SE	Df	*t*-Value	*p*-Value
Masked Lapwing	Mass (g)	0.043 ± 0.252	87	0.187	0.852
	Tarsus length (mm)	−0.153 ± 0.188	87	−0.815	0.417
	Tarsus plus toe length (mm)	−0.512 ± 0.335	87	−1.530	0.130
	Bill length (mm)	−0.209 ± 0.120	87	−1.738	0.086
	Head plus bill length (mm)	−0.274 ± 0.192	87	−1.423	0.158
	Body condition (SMI)	0.166 ± 0.237	87	0.701	0.701
	PC1 (mass and tarsus length)	0.087 ± 0.177	127	0.492	0.623
	PC2 (head size)	0.227 ± 0.176	127	1.290	0.199
Red-capped Plover	Mass (g)	−0.026 ± 0.079	25	−0.335	0.741
	Tarsus length (mm)	0.474 ± 0.145	25	3.264	**0.003**
	Tarsus plus toe length (mm)	0.356 ± 0.242	25	1.469	0.154
	Bill length (mm)	−0.054 ± 0.076	25	−0.718	0.479
	Head plus bill length (mm)	−0.038 ± 0.200	25	−0.188	0.853
	Body condition (SMI)	0.222 ± 0.350	25	0.633	0.533
	PC1 (head size)	−0.072 ± 0.149	30.092	−0.482	0.633
	PC2 (mass and tarsus length)	−0.314 ± 0.172	35.592	−1.829	0.076
Hooded Plover	Mass (g)	−0.201 ± 0.220	13	−0.916	0.376
	Tarsus length (mm)	0.041 ± 0.220	13	0.184	0.857
	Tarsus plus toe length (mm)	−0.136 ± 0.282	13	−0.481	0.638
	Bill length (mm)	0.095 ± 0.215	13	0.443	0.665
	Head plus bill length (mm)	−0.150 ± 0.312	13	−0.480	0.639
	Body condition (SMI)	−0.098 ± 0.363	13	−0.271	0.791
	PC1 (structural size)	0.159 ± 0.309	25.588	0.512	0.613

**Table 6:** Species-specific Linear Mixed-effect Models comparing chick growth (mass gained and tarsus length increase between captures) among the sexes. Significant values are emboldened.

Species	Metric	Variable	C ± SE	Df	*t*-Value	*p*-Value
Masked Lapwing	Mass (g)	Sex	21.606 ± 23.603	12	0.915	0.378
		Chick age (days)	2.530 ± 1.039	13	2.435	**0.030**
		Sex × chick age (days)	−0.412 ± 0.618	12	−0.667	0.517
	Tarsus length (mm)	Sex	2.763 ± 4.629	12	0.597	0.562
		Chick age (days)	0.329 ± 0.157	13	2.103	0.056
		Sex × chick age (days)	−0.047 ± 0.121	12	−0.387	0.706
Red-capped Plover	Mass (g)	Sex	4.169 ± 7.399	10	0.563	0.586
		Chick age (days)	0.590 ± 0.272	10	2.170	0.055
		Sex × chick age (days)	−0.264 ± 0.305	10	−0.864	0.408
	Tarsus length (mm)	Sex	2.951 ± 2.662	10	1.109	0.294
		Chick age (days)	0.214 ± 0.098	10	2.194	0.053
		Sex × chick age (days)	−0.129 ± 0.110	10	−1.173	0.268
Hooded Plover	Mass (g)	Sex	6.032 ± 12.617	5	0.478	0.653
		Chick age (days)	2.143 ± 0.333	28	6.439	**<0.001**
		Sex × chick age (days)	−0.265 ± 0.477	5	−0.556	0.602
	Tarsus length (mm)	Sex	2.613 ± 2.847	5	0.918	0.401
		Chick age (days)	0.231 ± 0.075	28	3.086	**0.005**
		Sex × chick age (days)	−0.098 ± 0.106	5	−0.931	0.395

**Table 7:** Species-specific Cox Proportional Hazard Regressions comparing survival between chick sexes for three species of shorebird. ‘brood size (one)’ is treated as the reference group for the categorical factor. Siblings of each radio-tracked Masked Lapwing chick were not individually identified as they died and only tracked lapwing individuals were analysed (one per brood); as such, a random effect of brood identity was not required in the Masked Lapwing model, and the output differed. Significant values are emboldened.

Species	Variable	C ± SE	Lower 95% CI	Upper 95%CI	X²	Df	*z*-Value	*p*-Value
Masked Lapwing	Sex	0.056 ± 0.337	0.547	2.050	-	1	0.171	0.865
	Brood size (two)	−0.205 ± 0.828	0.161	4.125	-	1	-0.248	0.804
	Brood size (three)	−0.179 ± 0.780	0.181	3.853	-	1	-0.230	0.818
	Brood size (four)	−0.348 ± 0.794	0.149	3.350	-	1	-0.438	0.661
	Brood size (five)	−0.444 ± 1.231	0.058	7.151	-	1	-0.361	0.718
Red-capped Plover	Sex	−0.436 ± 0.522	0.232	1.798	0.70	1	-	0.400
	Brood size (two)	1.365 ± 1.160	0.403	38.009	1.39	1	-	0.240
	Brood identity	-	-	-	62.32	32.21	-	**0.001**
Hooded Plover	Sex	0.430 ± 0.463	0.620	3.810	0.086	1	-	0.350
	Brood size (two)	0.308 ± 0.570	0.445	4.159	0.029	1	-	0.590
	Brood size (three)	0.565 ± 0.754	0.401	7.714	0.056	1	-	0.450
	Brood identity	-	-	-	19.57	10.80	-	**0.048**

## Appendix A

**Table A1:** Ordinal logistic model (Masked Lapwing) and binomial logistic model (Hooded Plover) examining variation in clutch size over the breeding season (separate models). A binomial linear model was used for the Hooded Plover because clutch sizes were binary (two and three eggs). Masked Lapwing clutches ranged between one and five eggs. Red-capped Plover is not modelled because clutch sizes of two eggs dominated throughout the season (96% of clutches).

Species	C ± SE	Df	*t*-Value	*z*-Value	*p*-Value
Masked Lapwing *	−0.008 ± 0.008	110	−1.009	-	0.313
Hooded Plover	−0.013 ± 0.015	25	-	−0.890	0.373

* Excluding the least frequent clutch sizes (one and five eggs) resulted in a *p*-value which approached, but did not meet our threshold in significance (*p* = 0.063).

**Table A2:** Study species, transmitter brand, length, width, depth, antenna length, mass, and operating life. All transmitters were black in colour.

Species	Chick Mass at Hatching (g; Mean ± SE)	Brand	Length (mm)	Width (mm)	Depth (mm)	Mass (g)	Operating Life (days)
Masked Lapwing	20.8 ± 0.3	Biotrack	16	7	4	1.05	133
Red-capped Plover	5.4 ± 0.1	Advanced Telemetry Systems	12	5	1.5	0.20	12
Hooded Plover	9.1 ± 0.2	Biotrack	15	6	3	0.45	34

**Table A3:** Species-specific morphometrics at hatching comparing chick mass, tarsus length, tarsus plus toe length, bill length and head plus bill length between the sexes. Means ± SE are provided.

Species	Metric	Males	Females	Sexes Combined
Masked Lapwing	Mass (g)	20.84 ± 0.21	20.84 ± 0.21	20.84 ± 0.15
	Tarsus length (mm)	30.01 ± 0.15	30.28 ± 0.15	30.14 ± 0.11
	Tarsus plus toe length (mm)	55.06 ± 0.30	55.48 ± 0.29	55.27 ± 0.21
	Bill length (mm)	11.96 ± 0.11	12.15 ± 0.11	12.06 ± 0.08
	Head plus bill length (mm)	35.75 ± 0.17	36.01 ± 0.16	35.88 ± 0.12
Red-capped Plover	Mass (g)	5.33 ± 0.07	5.32 ± 0.10	5.33 ± 0.06
	Tarsus length (mm)	19.47 ± 0.13	19.08 ± 0.14	19.28 ± 0.10
	Tarsus plus toe length (mm)	34.98 ± 0.24	34.64 ± 0.20	34.82 ± 0.16
	Bill length (mm)	7.13 ± 0.07	7.30 ± 0.11	7.21 ± 0.06
	Head plus bill length (mm)	23.40 ± 0.19	23.57 ± 0.17	23.48 ± 0.13
Hooded Plover	Mass (g)	9.20 ± 0.15	9.19 ± 0.17	9.20 ± 0.11
	Tarsus length (mm)	20.17 ± 0.16	20.20 ± 0.15	20.18 ± 0.11
	Tarsus plus toe length (mm)	39.28 ± 0.28	39.59 ± 0.17	39.45 ± 0.16
	Bill length (mm)	8.32 ± 0.16	8.07 ± 0.13	8.19 ± 0.10
	Head plus bill length (mm)	27.48 ± 0.30	27.27 ± 0.18	27.37 ± 0.17
